# Early administration of remdesivir plus convalescent plasma therapy is effective to treat COVID-19 pneumonia in B-cell depleted patients with hematological malignancies

**DOI:** 10.1007/s00277-022-04924-6

**Published:** 2022-07-14

**Authors:** Ferenc Magyari, László Imre Pinczés, Edit Páyer, Katalin Farkas, Szilvia Ujfalusi, Ágnes Diószegi, Máté Sik, Zsófia Simon, Gergely Nagy, Zsuzsanna Hevessy, Béla Nagy, Árpád Illés

**Affiliations:** 1 Division of Hematology, Department of Internal Medicine, Faculty of Medicine, University of Debrecen, Debrecen, Hungary; 2 Doctoral School of Clinical Medicine, University of Debrecen, Debrecen, Hungary; 3 Division of Endocrinology, Department of Internal Medicine, Faculty of Medicine, University of Debrecen, Debrecen, Hungary; 4 Department of Radiology, Faculty of Medicine, University of Debrecen, Debrecen, Hungary; 5 Department of Emergency Medicine, Faculty of Medicine, University of Debrecen, Debrecen, Hungary; 6 Department of Laboratory Medicine, Faculty of Medicine, University of Debrecen, Debrecen, Hungary

**Keywords:** COVID-19, SARS-CoV-2, Convalescent plasma treatment, Remdesivir, Hematological malignancies, Immunodeficiency

## Abstract

Patients with hematological malignancies (HMs) are at a higher risk of developing severe form and protracted course of COVID-19 disease. We investigated whether the combination of viral replication inhibition with remdesivir and administration of anti-SARS-CoV-2 immunoglobulins with convalescent plasma (CP) therapy might be sufficient to treat B-cell-depleted patients with COVID-19. We enrolled 20 consecutive patients with various HMs with profound B-cell lymphopenia and COVID-19 pneumonia between December 2020 and May 2021. All patients demonstrated undetectable baseline anti-SARS-CoV-2 immunoglobulin levels before CP. Each patient received at least a complete course of remdesivir and at least one unit of CP. Previous anti-CD20 therapy resulted in a more prolonged SARS-CoV-2 PCR positivity compared to other causes of B-cell lymphopenia (*p* = 0.004). Timing of CP therapy showed a significant impact on the clinical outcome. Simultaneous use of remdesivir and CP reduced time period for oxygen weaning after diagnosis (*p* = 0.017), length of hospital stay (*p* = 0.007), and PCR positivity (*p* = 0.012) compared to patients who received remdesivir and CP consecutively. In addition, time from the diagnosis to CP therapy affected the length of oxygen dependency (*p* < 0.001) and hospital stay (*p* < 0.0001). In those cases where there were at least 10 days from the diagnosis to plasma administration, oxygen dependency was prolonged vs. patients with shorter interval (*p* = 0.006). In conclusion, the combination of inhibition of viral replication with passive immunization was proved to be efficient and safe. Our results suggest the clear benefit of early, combined administration of remdesivir and CP to avoid protracted COVID-19 disease among patients with HMs and B-cell lymphopenia.

**Supplementary Information:**

The online version contains supplementary material available at 10.1007/s00277-022-04924-6.

## Introduction

The newly identified coronavirus disease 2019 (COVID-19), caused by severe acute respiratory syndrome coronavirus 2 (SARS‐CoV‐2), has become a global pandemic characterized by severe atypical pneumonia in 15–20% of all cases and a rapid human‐to‐human transmission. Immunocompromised patients, especially patients with hematological malignancies (HMs), are at a high risk of developing a severe form and protracted course of COVID-19 [1–3]. HMs are associated with defects in humoral and cellular immunity, while treatment often exacerbates these immune deficiencies and can lead to prolonged B-cell depletion, contributing to unfavorable COVID-19 outcomes [4].

Remdesivir, an inhibitor of the viral RNA-dependent RNA polymerase, was identified early as a promising therapeutic candidate for COVID-19 because of its ability to inhibit SARS-CoV-2 [5]. However, antiviral monotherapy appears to be an insufficient treatment option in the absence of humoral immunity [6–8]. Convalescent plasma (CP) isolated from patients who have recovered from COVID-19 infection contains high levels of antibodies against SARS-CoV-2 that may be suitable for passive immunization of recipients for both prophylactic and therapeutic purposes [9]. The effectiveness of CP transfusion in other infectious diseases has been demonstrated over the past decades [10, 11]. The use of CP in COVID-19 patients has shown conflicting results and only a limited amount of data is available on its administration in B-cell-depleted patients [12–14]. The use of CP has not been specifically assessed in clinical trials in the population of B-cell depleted patients. To avoid the development of protracted COVID-19 disease, it is of paramount importance in HM patients. In addition to the cumulative infectious complications and deterioration in quality of life, the mortality of patients due to the underlying disease is a major concern. Dose reduction or postponement of treatment due to COVID-19 can lead to reduced overall- and progression-free survival of HM patients with active disease.

In this study, we hypothesize that the combination of exogenous anti-SARS-CoV-2 antibodies and inhibition of viral replication may be sufficient to effectively treat COVID-19 patients with different HMs. Therefore, we investigated the clinical and laboratory response to the combination of remdesivir and CP in a considerably large cohort of B-cell-depleted subjects.

## Methods

### Subjects

The observational, single-center study was conducted in the COVID-19 Epidemiological Care Centre, University of Debrecen, Hungary, between December 2020 and July 2021. Diagnosis of COVID-19 was based on a positive PCR test result for SARS-CoV-2 infection from a nasopharyngeal swab. Evaluated patients demonstrated a profound B-cell lymphopenia based on flow cytometry analysis of peripheral blood lymphocyte subpopulations. They showed undetectable baseline anti-SARS-CoV-2 immunoglobulin (Ig) levels before CP transfusion according to the results of automated anti-SARS-CoV-2 nucleocapsid- and Spike protein1-receptor binding domain (S1-RBD)-specific total Ig tests. Each patient received a complete course of remdesivir and at least one unit (200 mL) of AB0 compatible CP during their treatment for COVID-19. Post-transfusion anti-SARS-CoV-2 Ig levels were measured 12 h after CP administration and regularly thereafter. Our research was undertaken in accordance with relevant guidelines and regulations. All patients involved signed an informed consent form. The severity of COVID-19 was evaluated according to the World Health Organization (WHO) Clinical Progression Scale [15].

### Data sources and definitions

Data were extracted from patients’ medical charts and were obtained on their demographics, complete medical history, comorbidities, detailed chemotherapy use, and malignancy status at admission. COVID-19-related data included time of symptom onset, diagnosis, laboratory and imaging results, specific medications, and oxygen supply. Remdesivir and CP therapy were considered simultaneous if CP therapy was initiated before completing the first cycle of remdesivir. Length of hospital stay was defined as the time period between the first admission for COVID-19 symptoms and final discharge or death.

### Treatment protocols

Remdesivir treatment was administered at a maintenance dose of 100 mg per day following a saturating dose of 200 mg intravenously. A treatment cycle consisted of 5–15 days. A patient was allowed a maximum of 2 treatment cycles. Absolute contraindications to remdesivir treatment were severe renal failure (GFR-EPI less than 30 mLl/min/1.73 m^2^) and acute liver failure (Child–Pugh Class C).

Plasma donors who recovered from COVID-19 infection signed a consent form for CP donation. The age of eligibility for donation was generally defined as between 18 and 60 years. Eligibility was restricted to those who were negative for human immunodeficiency virus (HIV), hepatitis B, hepatitis C, and syphilis. Donors were free of any symptoms and complaints for at least 2 weeks at pre-screening, were negative for SARS-CoV-2 PCR test on a nasopharyngeal swab, and had detectable serum anti-SARS-CoV-2 IgG level. The donors underwent plasmapheresis providing 600 mLl of plasma collected over approximately 1 h. The final product was irradiated and stored by the Hungarian National Blood Transfusion Service. The preparations were dispensed in 3 × 200 mL fractions. CP was derived from patients cured of COVID-19 in the same wave (alpha and beta mutant, 2nd and 3rd wave, from November 2020 to June 2021). Via the monitoring protocol, antibody measurements were performed immediately after CP administration, and at 12, 48, 72, and 138 h after CP infusion.

### Laboratory analysis

Total Ig levels against SARS-CoV-2 nucleocapsid and S1-RBD were measured by automated immunoassays (Elecsys® Anti-SARS-CoV-2 and Anti-SARS-CoV-2 S tests, Roche Diagnostics, Mannheim, Germany) at the cut-off value of 1.0 (cut-off index, COI) and 0.8 U/mL titer, respectively, according to the instructions of the manufacturer.

B-cell depletion was defined by flow cytometry before and under CP plus remdesivir therapy to analyze whether peripheral CD19 + B cells were ≤ 80/μLl or percentage among total lymphocytes were ≤ 5%. White blood cells were stained by anti-CD19-PE and anti-CD3-APC (Becton Dickinson, Franklin Lakes, NJ, USA). In case of anti-CD20 therapy, 500,000 dual-color–labeled leukocytes were acquired on a FASCanto II flow cytometer (Becton Dickinson, Franklin Lakes, NJ, USA), while 100,000 events were analyzed in the follow-up samples. Results were expressed as the percentage of lymphocytes.

### Statistical analysis

Categorical variables are given as their frequencies and percentages, while continuous variables with medians and ranges. Continuous variables were evaluated using the Mann–Whitney *U* test or *t*-test based on the normality of the data. The Shapiro–Wilk test was used for evaluation of data normality. Simple linear regression was used to test if the time interval from COVID-19 diagnosis to CP therapy or post-transfusion anti-SARS-CoV-2 Ig titers predicted the outcome. The level of statistical significance was considered at *p* < 0.05. Statistical analyses were performed using SPSS 26.0 (IBM Corp., Armonk, NY, USA).

## Results

A total of 20 B-cell depleted hematological patients were included in our study (Table 1, Online Resource 1). The median age at COVID-19 diagnosis was 56 years (range 27–76) with a male predominance (male to female ratio 1.8). All patients had biopsy-proven HM with the majority suffering from an active disease. Sixteen (80%) patients had B-cell origin disorder; only a minority of them was heavily pre-treated. Thirteen (65%) subjects received anti-CD20 therapy within the last 2 years, while only two (10%) patients were treatment-naive at the time of COVID-19 diagnosis. None of the vaccinated patients had detectable baseline anti-SARS-CoV-2 Ig levels at the time of diagnosis.

**Table 1:** Patient characteristics

Characteristics	Data
Age, median, years (range)	56 (27–76)
Male/female, *n*	13/7
Hematological malignancy, *n* (%)	
Acute lymphoid leukemia	1 (5)
Acute myeloid leukemia	2 (10)
Mixed phenotype acute leukemia	1 (5)
Chronic lymphoid leukemia	4 (20)
Diffuse large B-cell lymphoma	4 (20)
Follicular lymphoma	2 (10)
Mantle cell lymphoma	3 (15)
Splenic marginal zone lymphoma	1 (5)
Myelofibrosis	1 (5)
Multiple myeloma	1 (5)
Disease status, *n* (%)	
Complete remission	9 (45)
Partial remission	3 (15)
Progressive disease	8 (40)
Previous lines of therapy, median, *n* (range)	1 (0–5)
Previous treatment with anti-CD20 therapy, *n* (%)	
Rituximab	10 (50)
Obinutuzumab	3 (15)
None	7 (35)
Last chemotherapy, *n* (%)	
Anti-CD20 + chemotherapy	3 (15)
Anti-CD20 maintenance	5 (25)
Classical chemotherapy	4 (20)
Small molecule	4 (20)
Treatment naive	2 (10)
Other	2 (10)
Hemopoietic stem cell transplantation, *n* (%)	
Autologous	5 (25)
Allogenic	1 (5)
None	14 (70)
COVID-19 severity score (WHO), *n* (%)	
4	8 (40)
5	10 (50)
6–7	0
8–9	2 (10)
COVID-19 specific treatments, *n* (%)	
Favipiravir	9 (45)
Dexamethasone	20 (100)
Tocilizumab	0
Bamlanivimab	5 (25)
Days of remdesivir therapy	
All patients, median, *n* (range) Short course (8–10 days), *n* (%) Intermediate course (11–20 days), *n* (%) Long course (21–30 days), *n* (%)	12.5 (8–30) 4 (20) 14 (70) 2 (10)
Convalescent plasma units, median, *n* (range)	4 (1–15)
Simultaneous use of remdesivir and CP, *n* (%)	17 (85)
COVID-19 vaccination, *n* (%)	2 (10)
Time from…, median, days (range)	
Symptoms onset to PCR positivity	3.5 (0–10)
Symptoms onset to antiviral therapy	6.5 (1–21)
Symptoms onset to CP therapy	13.5 (3–44)
PCR positivity to antiviral therapy	1 (0–20)
PCR positivity to CP therapy	7.5 (2–44)
PCR positivity to discharge (*n* = 19)	22 (10–69)
PCR positivity to PCR negativity (*n* = 19)	63 (6–204)
CP therapy to loss of oxygen dependency (*n* = 12)	9.5 (2–36)
CP therapy to discharge (*n* = 19)	16 (4–55)
CP therapy to PCR negativity (*n* = 19)	59 (4–188)
Laboratory parameters	
Absolute neutrophil count, median, G/L (range)	2.82 (0.27–9.25)
Absolute lymphocyte count, median, G/L (range)	0.52 (0–5.44)
CD19 + B-cell count, cell/µL (range)	0 (0–77.7)
CD4 + T-cell count, cell/µL (range)	112 (18–1029)
CD8 + T-cell count, cell/µL (range)	165 (5–501)
IgG, median, g/L (range)	7.5 (0–14)
IgA, median, g/L (range)	0.8 (0–2.3)
IgM median, g/L (range)	0.21 (0–1.98)

Small molecules include ibrutinib monotherapy (*n* = 3) and venetoclax plus acalabrutinib (*n* = 1). Other therapies include: venetoclax plus Obinutuzumab (*n* = 1) and ruxolitinib (*n* = 1)

*WHO* World Health Organization, *CP* convalescent plasma, *PCR* polymerase chain reaction, *G* giga, *L* liter, *g* gram, *µ* micro, *Ig* immunoglobulin

Most patients were diagnosed and admitted early with COVID-19, with a median of 3.5 days after the onset of symptoms. Most of them were administered antiviral treatment immediately, within 24 h after hospital admission. Eighteen (90%) patients experienced a moderate COVID-19 disease course according to the WHO Severity Score (4–5). Median days of remdesivir treatment and median units of CP therapy were 12.5 (8–30) and 4 (1–15), respectively. Remdesivir and CP were administered simultaneously in case of 17 patients. Five patients (25%) received a repeated treatment cycle of remdesivir. Apart from dexamethasone, CP, and remdesivir, COVID-19-specific treatments were administered after initial therapy with an intent to accelerate viral clearance. Five patients received bamlanivimab treatment after clinical resolution of COVID-19 symptoms, but persisting PCR positivity, 24–98 days after initial diagnosis.

**Fig. 1:**
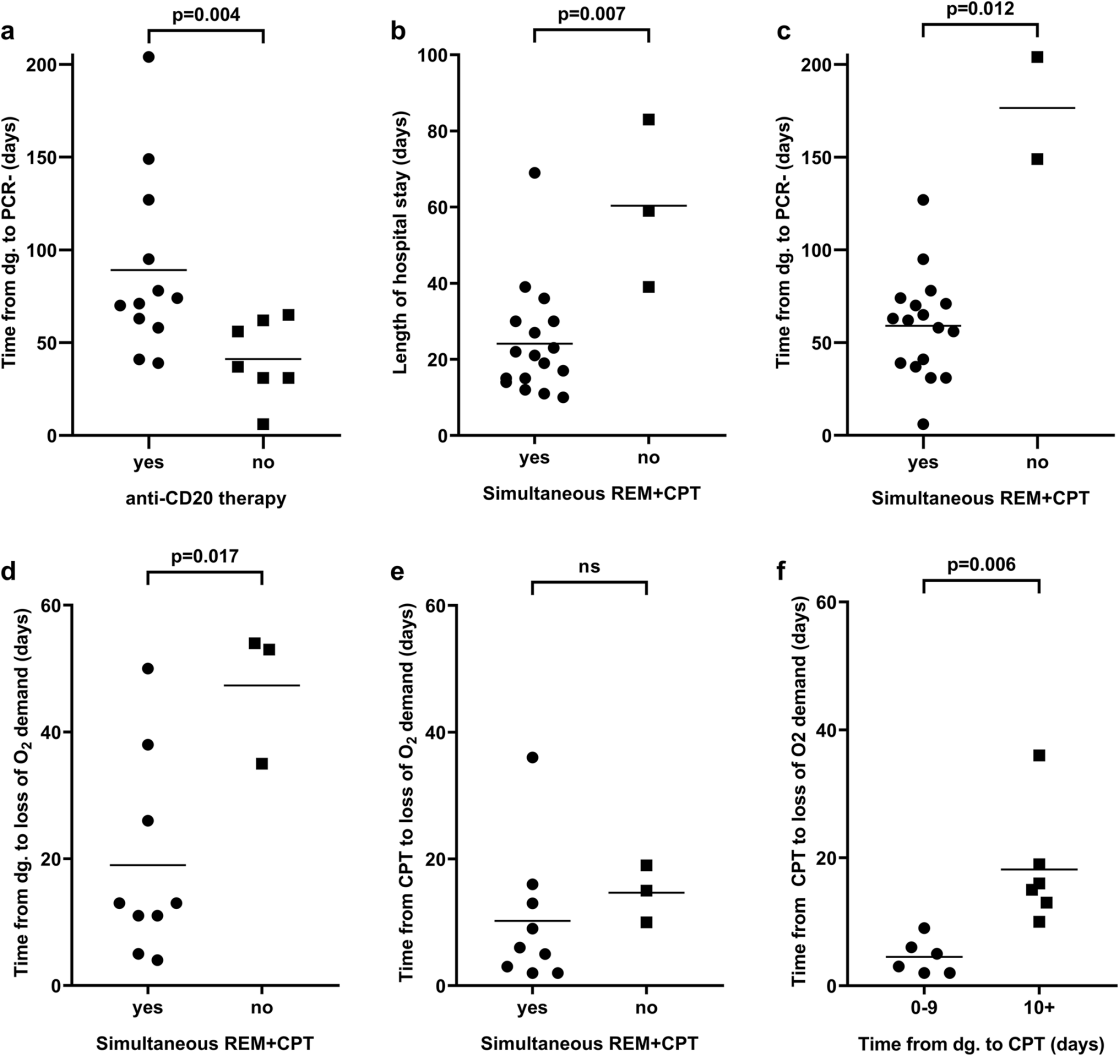
The effect of clinical parameters and timing of convalescent plasma therapy on outcome. Anti-CD20 therapy prior to COVID-19 diagnosis was associated with a prolonged PCR positivity (**a**). Subjects receiving remdesivir and CP (REM + CPT) simultaneously were discharged earlier compared to patients treated consecutively (**b**). Simultaneously treated patients showed a remarkably reduced time interval to PCR negativity (**c**), loss of oxygen demand from diagnosis (**d**), while there was no difference in the length of oxygen therapy after initiation of CP therapy (**e**). Early CP administration led to an early cessation of oxygen demand, compared to patients receiving CP at least 10 days after COVID-19 diagnosis (**f**). Comparisons were performed using Mann–Whitney *U*-test or *t*-test. PCR polymerase chain reaction, dg. diagnosis, REM remdesivir, CPT convalescent plasma therapy, O_2_ oxygen

Anti-CD20 therapy prior to COVID-19 diagnosis resulted in a prolonged PCR positivity compared to non-recipients (*p* = 0.004) (Fig. 1a). The severity of B-cell depletion, disease status, other treatment modalities, and demographic parameters did not affect the outcome of these patients. We analyzed data on T-cell counts (CD3 + , CD4 + , CD8 + , CD56 + /CD3 − , CD56 + /CD3 + cell populations) at COVID-19 diagnosis but did not find noteworthy correlation with any of the observed outcomes in this study.

**Fig. 2:**
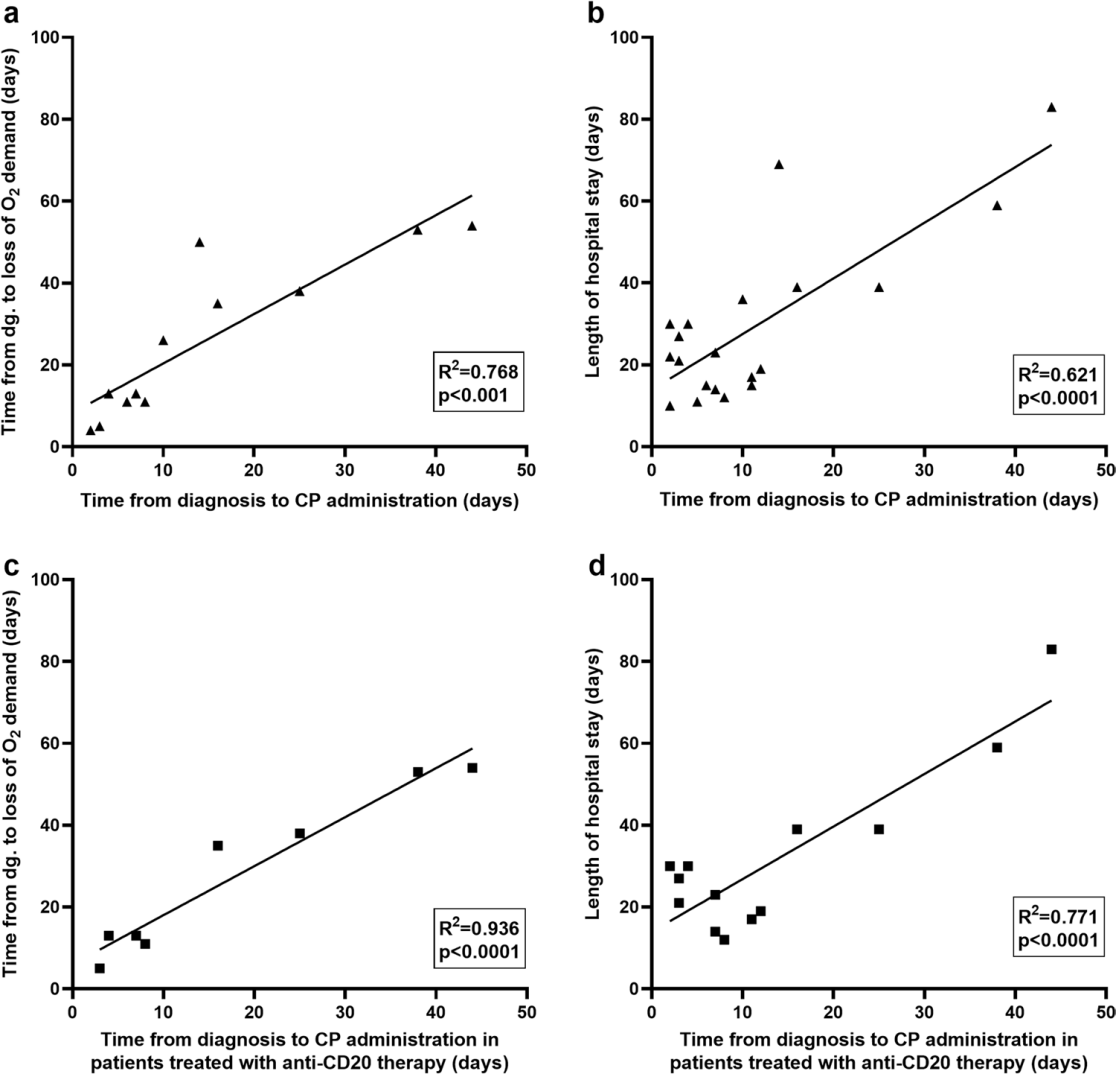
Correlation between the timing of convalescent plasma therapy and outcome. Based on linear regression analysis, there was a statistically significant relationship between the time period from diagnosis to CP therapy and the length of oxygen dependency as well as the length of hospital stay in all patients (**a**, **b**), and in the anti-CD20 treatment group (**c**, **d**). O_2_ oxygen, dg. diagnosis, CP convalescent plasma

Three patients (with follicular lymphoma, diffuse large B-cell lymphoma transformed from follicular lymphoma, and acute myeloid leukemia) received remdesivir monotherapy before CP and showed a temporary clinical improvement but relapsed within a few days after completion of the antiviral treatment. However, all patients simultaneously receiving a combination of remdesivir and CP experienced a fast and sustained resolution of symptoms. The median length of hospital stay was 22.5 days (10–83), while patients receiving remdesivir and CP simultaneously were discharged earlier compared to patients treated consecutively (*p* = 0.007) (Fig. 1b). Simultaneously treated patients also experienced a remarkably reduced time to PCR negativity (*p* = 0.012) and loss of oxygen demand (*p* = 0.017), while there was no difference in the length of oxygen therapy after initiation of CP therapy (Fig. 1c–e). Furthermore, early CP administration led to an early cessation of oxygen demand, compared to patients receiving CP at least 10 days after COVID-19 diagnosis (*p* = 0.006) (Fig. 1f). With linear regression analysis, time from diagnosis to CP therapy showed a statistically significant impact on the length of oxygen dependency (R^2^ = 0.768, *F* = 33.1, *p* < 0.001) and on the length of hospital stay (*R*^2^ = 0.621, *F* = 29.4, *p* < 0.0001) (Fig. 2a and b). This correlation was even more pronounced in patients receiving anti-CD20 treatment (R^2^ = 0.936, *F* = 87.6, *p* < 0.0001 and R^2^ = 0.771, *F* = 36.9, *p* < 0.0001, respectively) (Fig. 2c and d).

**Fig. 3:**
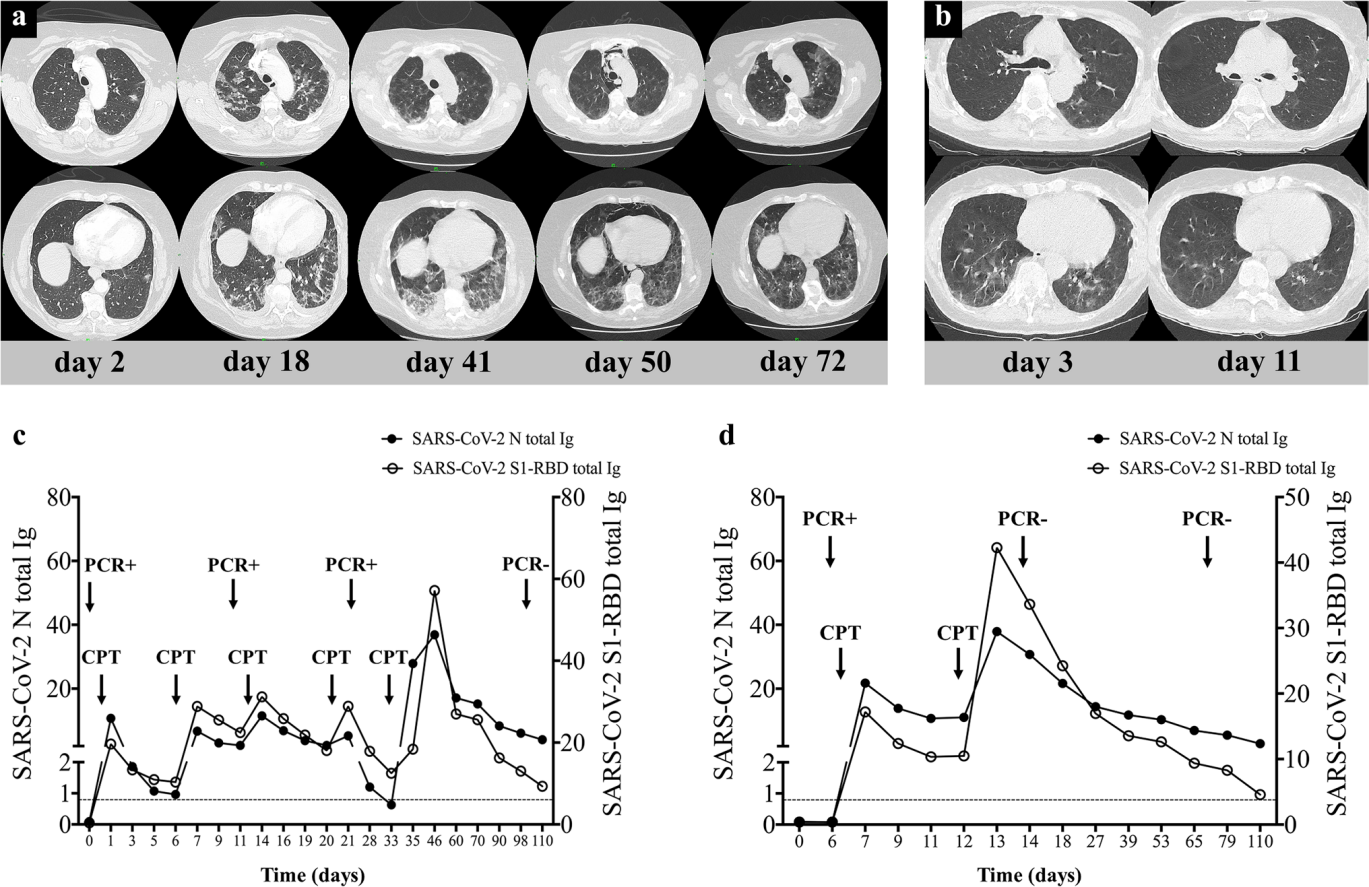
Summary and comparison of the clinical course of B-cell depleted patients with protracted (**a**, **c**) and fast-responding (**b**, **d**) COVID-19 pneumonia. Representative CT-scan images and SARS-CoV-2 total Ig levels monitored through the length of hospital stay of two patients. Patient 1 suffered from protracted COVID-19 pneumonia (**a**, **c**), while patient 2 had a good response to treatment showing a faster recovery (**b**, **d**). A gradual decrease in antibody consumption in our patients receiving multiple CP transfusions was observed that strongly correlated with clinical and radiological improvement. Patient 1 and patient 2 had follicular lymphoma, and multiple myeloma, respectively. Ig immunoglobulin, PCR polymerase chain reaction, CPT convalescent plasma therapy

All patients presented detectable anti-SARS-CoV-2 IgG 12 h after the first CP transfusion. Anti-SARS-CoV-2 nucleocapsid- and S1-RBD-specific total Ig levels were median 10.7 (0.3–30.2) COI and 17.2 U/mL (2.8–31.0) after the first, while 12.2 (2.8–37.9) COI and 24.6 U/mL (8.4–126.1) after the second dose of CP therapy, respectively. A weak negative correlation was observed between the anti-SARS-CoV-2 nucleocapsid-specific total Ig level after the second transfusion and the time from transfusion to discharge (*R*^2^ = 0.312, *F* = 6.8, *p* = 0.02) (data not shown). Interestingly, other post-transfusion anti-SARS-CoV-2 Ig levels did not affect the time interval from CP therapy to oxygen weaning, discharge, or PCR positivity. We experienced a gradual decrease in antibody consumption in our patients receiving multiple CP transfusions that was strongly correlated and concurrent with clinical and radiological improvement (Fig. 3).

Finally, no COVID-19-related death was recorded. One patient died as a result of lymphoma progression. He received remdesivir and CP consecutively and was asymptomatic for COVID-19 at death with a persistent SARS-CoV-2 PCR positivity. No transfusion-related adverse events were observed during or after CP therapy.

## Discussion

Several groups have recently reported a protracted disease course of COVID-19 in B-cell-depleted patients [16–21]. A common phenomenon was a transient improvement during antiviral therapy and a rapid relapse after the conclusion of treatment. Also, a recurrent experience was a sustained improvement following CP therapy, occasionally with cessation of oxygen dependency within days. As we have had similar experiences in our own patient population with remdesivir monotherapy, we hypothesized that inhibition of viral replication in the absence of an effective humoral immune response was not sufficient for recovery from COVID-19. As part of the humoral immune response, antibodies produced by B-cells are essential for antibody-driven virus elimination. Also, accumulating evidence suggested that specific T-cell responses are unable to control SARS-CoV-2 viral infection in the absence of humoral immunity and neutralizing antibodies [1, 6, 22]. These observations led to the administration of remdesivir in combination with CP in such patient cohorts.

This study reports the clinical benefit of the combination of remdesivir and CP therapy in 20 consecutive patients with COVID-19 disease and B-cell lymphopenia, considered as a consequence of either the underlying disease or the treatment given for it. While COVID-19-specific antiviral treatments can induce a transient decrease in symptoms, they fail to produce a complete viral clearance and sustained clinical improvement in this patient population. In our series, former treatment with anti-CD20 monoclonal antibodies was associated with prolonged SARS-CoV-2 PCR positivity, which can be attributed to the extended and profound B-cell depletion caused by the compound with an elevated risk of protracted viral shedding and within-host viral evolution (Online Resource 2) [23]. Also, achievement of early SARS-CoV-2 PCR negativity, and thus avoiding delay in treatment, is of particular importance for patients who acquired COVID-19 in the setting of a progressing HM [24].

For the same reason, the rapid and sustained improvement in the clinical condition of immunocompromised hematological patients is of paramount importance. Our results provide a clear evidence that this population can benefit from early and combined administration of remdesivir and CP. Concomitant use showed benefits regarding hospital stay, length of oxygen dependency, and time needed for PCR negativity suggesting early treatment initiation as the most important aspect. The fact that there was no difference in the length of oxygen demand after plasma administration between patients receiving remdesivir and CP treatment at the same time and those receiving it separately implies that passively transferred antibodies play a key role in COVID-19 clinical recovery. The overall clinical improvement observed after CP therapy correlated strongly with early administration, as demonstrated by the rapid decrease in oxygen demand and length of hospital stay. The decreasing effectiveness of plasma therapy late in the course of the disease or even after remdesivir therapy has been also emphasized by a recent large-scale study [25]. Our observation is consistent with previously published reports regarding the discrepancy between the time needed for clinical recovery and PCR negativity. In the face of the rapidity of clinical response, time to viral clearance after passive immunotherapy is highly variable in B-cell-depleted patients [8].

As Gharbharan et al. reported, the peak and duration of immediate seropositivity after CP transfusion varied substantially [26]. We assume that this phenomenon may be the result of the individual consumption rate of SARS-CoV-2 IgG depending on the extent and severity of COVID-19. We experienced a gradual decrease in antibody consumption in our patients receiving multiple CP transfusions that strongly correlated with clinical and radiological improvement. Also, while the specificities of the Hungarian blood banking system and the paucity of available supply sometimes resulted in a delay of CP transfusion, we were able to utilize this observation, and tailor CP therapy to the rate of decline in anti-SARS-CoV-2 Ig titers [27]. With subsequent CP transfusions, we aimed to achieve the rate of decline observed in the immunocompetent patient population with moderate-severe COVID-19 disease and experienced a spectacular clinical improvement when this rate was approached [28]. Importantly, we did not allow SARS-CoV-2 Ig levels to fall below any of their cut-off values. The strength of our strategy is supported by observations that post-infection elimination of SARS-CoV-2 IgG predisposes patients to early relapse [16, 29]. Unlike Rodionov et al., we cannot report on a strong correlation between anti-SARS-CoV IgG titers and improvement in clinical status [30]. While the WHO Clinical Progression Scale was not monitored on a daily basis, we considered cessation of oxygen demand and discharge from hospital to be strong indicators of clinical improvement. In summary, our data suggest that the rate and magnitude of clinical improvement are in relation to maintenance of Ig levels above the cut-off levels, preventing rapid decline through repeated CP transfusions.

Hyperimmune intravenous immunoglobulins derived from plasma and monoclonal antibodies against the structural proteins of SARS-CoV-2 are tempting treatment options in B-cell-depleted patients with COVID-19 but face permanent competition from CP. The amount of plasma needed to produce a single dose of hyperimmune globulin raises ethical concerns during a pandemic. Specific monoclonal antibodies are recommended both for non-hospitalized patients with mild to moderate COVID-19 who are at high risk of developing severe illness, including those immunocompromised due to age, medical conditions, or immunosuppressive medications, and for hospitalized patients with moderate to severe disease (not requiring high-flow oxygen, non-invasive, or mechanical ventilation) and negative SARS-CoV-2 anti-spike IgG. However, the antibodies developed may lose effectiveness when a new mutant becomes dominant, and the safety and efficacy of combining them with remdesivir are unknown. With the rapid emergence of SARS-CoV-2 variants, therapeutic gaps may be needed to be filled while production and deployment of refined monoclonal antibodies occur. In this context, CP treatment will certainly be inevitable until the global pandemic is over.

Effective patient education may be the reason why patients were admitted with early symptoms and mostly mild-to-moderate COVID-19 severity. This may have contributed to our excellent survival rate, which is far superior to the data described in multicenter analyses [2, 13, 31]. Our therapeutic approach also appears to be effective during pregnancy with no evidence of fetal distress [32, 33].

We acknowledge the limitations of this non-randomized study without control group comparisons. However, in our opinion, real-world data is of particular importance in patient populations with unusual disease settings and thus limited possibilities for performing a sufficiently powered randomized control trial. To adequately attribute any outcome to the combination of remdesivir and CP therapy, further information needs to be acquired in this clinical setting.

In conclusion, we reported the outcomes of 20 B-cell depleted patients with HMs and COVID-19 infection. Our results support the assumption that the early combination of antiviral therapy and passive immunotherapy facilitates clinical recovery from COVID-19 and viral clearance of SARS-CoV-2. Passive transfer of COVID-19-specific neutralizing antibodies with CP therapy was effective and safe in a patient population with compromised immune systems, irrespective of the cause resulting in B-cell lymphopenia.

## Supplementary Information

Below is the link to the electronic supplementary material.

Supplementary file1 (PDF 339 KB)

## Data Availability

The datasets generated during and/or analyzed during the current study are available from the corresponding author on reasonable request.
