# Supplementary material for: Early administration of remdesivir plus convalescent plasma therapy is effective to treat COVID-19 pneumonia in B-cell depleted patients with hematological malignancies
Source: Ann Hematol. 2022 Jul 14;101(10):2337–45. doi: 10.1007/s00277-022-04924-6 (PMC9282831; doi:10.1007/s00277-022-04924-6)
Supplement: Supplementary file 1 — Supplementary file1 (PDF 339 KB) [file 277_2022_4924_MOESM1_ESM.pdf]

Article title

Early administration of remdesivir plus convalescent plasma therapy is effective to treat COVID-19 pneumonia in B-cell depleted patients with hematological malignancies

Journal name

Annals of Hematology

Author names

[1,2,#] Ferenc Magyari, [1,2,#] László Imre Pinczés, [1,2] Edit Páyer, [1,2] Katalin Farkas, [3] Szilvia Ujfalusi, [3] Ágnes Diószegi, [4] Máté Sik, [1] Zsófia Simon, [5] Gergely Nagy, [6] Zsuzsanna Hevessy, [6,&] Béla Nagy Jr., [1,2,&] Árpád Illés

[#] These first authors equally contributed to this work.

[&] These last authors equally contributed to this work.

Affiliation

[1] Division of Hematology, Department of Internal Medicine, Faculty of Medicine, University of Debrecen, Debrecen, Hungary; [2] Doctoral School of Clinical Medicine, University of Debrecen, Debrecen, Hungary; [3] Division of Endocrinology, Department of Internal Medicine, Faculty of Medicine, University of Debrecen, Debrecen, Hungary; [4] Department of Radiology, Faculty of Medicine, University of Debrecen, Debrecen, Hungary; [5] Department of Emergency Medicine, Faculty of Medicine, University of Debrecen, Debrecen, Hungary; [6] Department of Laboratory Medicine, Faculty of Medicine, University of Debrecen, Debrecen, Hungary

Corresponding author

László Imre Pinczés pinczes.laszlo.imre@med.unideb.hu

	ANC (G/L)	ALC (G/L)	IgG (g/L)	IgA (g/L)	IgM (g/L)	CD3+ (cell/uL)	CD4+ (cell/uL)	CD8+ (cell/uL)	CD4/CD8 (ratio)	CD56+/CD3- (cell/uL)	CD19+ (cell/uL)
**Patient #1**	2.32	0.56	7.87	0.61	0	348	67	277	0.24	26	0
**Patient #2**	2.01	0	7.5	0.8	0.26	273	169	101	1.67	222	0
**Patient #3**	0.81	0	0	0	0	371	265	102	2.60	34	77.7
Patient #4	1.84	0.84	4.61	0	0.33	105	42	62	0.67	16	0
**Patient #5**	7.4	1.34	2.04	0.16	0.17	537	165	339	0.49	102	0
**Patient #6**	1.19	0.80	2.61	0	0	538	255	282	0.90	16	0
Patient #7	1.75	0.93	n/a	n/a	n/a	455	309	128	2.41	18	21
Patient #8	5.87	0.15	11.5	0	0	108	43	58	0.74	15	23
**Patient #9**	3.31	0.24	10.00	0	1.1	23	18	5	3.81	6	0
**Patient #10**	4.35	0.41	n/a	n/a	n/a	187	53	133	0.39	26	0
**Patient #11**	9.25	0.37	3.30	0	0	215	55	159	0.35	3	0
Patient #12	4.15	5.44	8.63	1.62	0.21	1613	1029	501	2.060	250	51.7
**Patient #13**	0.38	0.48	13.9	2.3	0.55	508	68	388	0.17	24	0
**Patient #14**	3.88	1.11	4.03	0.97	0	297	111	171	0.65	101	28.7
Patient #15	8.83	0.34	14.1	1.8	1.98	306	188	106	1.78	87	9
Patient #16	0.27	0	n/a	n/a	n/a	1125	673	385	1.75	193	0
**Patient #17**	2.09	0.47	7.8	0.8	0.49	85	41	43	0.94	33	6.7
**Patient #18**	3.62	0.73	10.8	1.3	0.64	499	243	231	1.050	68	0
Patient #19	3.92	1.93	5.19	1.14	0.38	382	104	243	0.43	20.00	9
**Patient #20**	1.06	0.89	7.40	0.90	0	591	113	471	0.24	24	0
**Normal value**	1.68-8	0.9-3.1	7-16	0.7-4	0.4-2.3	550-1800	310-1150	140-850	-	80-690	< 80
**All patients**											
Median	2.815	0.52	7.5	0.8	0.21	359.5	112	165	0.82	26	0
Min	0.27	0	0	0	0	23	18	5	0.17	3	0
Max	9.25	5.44	14.1	2.3	1.98	1613	1029	501	3.81	250	77.7
**Patients receiving anti-CD20 therapy**											
Median	2.32	0.48	7.45	0.705	0.085	348	111	171	0.65	26	0
Min	0.38	0	0	0	0	23	18	5	0.17	3	0
Max	9.25	1.34	13.9	2.3	1.1	591	265	471	3.81	222	77.7
**Patients without anti-CD20 therapy**											
Median	3.92	0.84	8.63	1.14	0.33	382	188	128	1.75	20	9
Min	0.27	0	4.61	0	0	105	42	58	0.43	15	0
Max	8.83	5.44	14.1	1.8	1.98	1613	1029	501	2.41	250	51.7

Online Resource 1 Baseline absolute neutrophil and lymphocyte counts, immunoglobulin G, A and M levels, T-cell (CD3+, CD4+, CD8+, CD4+/CD8+), NK-cell (CD56+/CD3-) and B-cell (CD19+) compartments of the peripheral blood at COVID-19 diagnosis. Data of anti-CD20 exposed patients is indicated in bold.

Abbreviations: ANC, absolute neutrophil count; ALC, absolute lymphocyte count; Ig, immunoglobulin

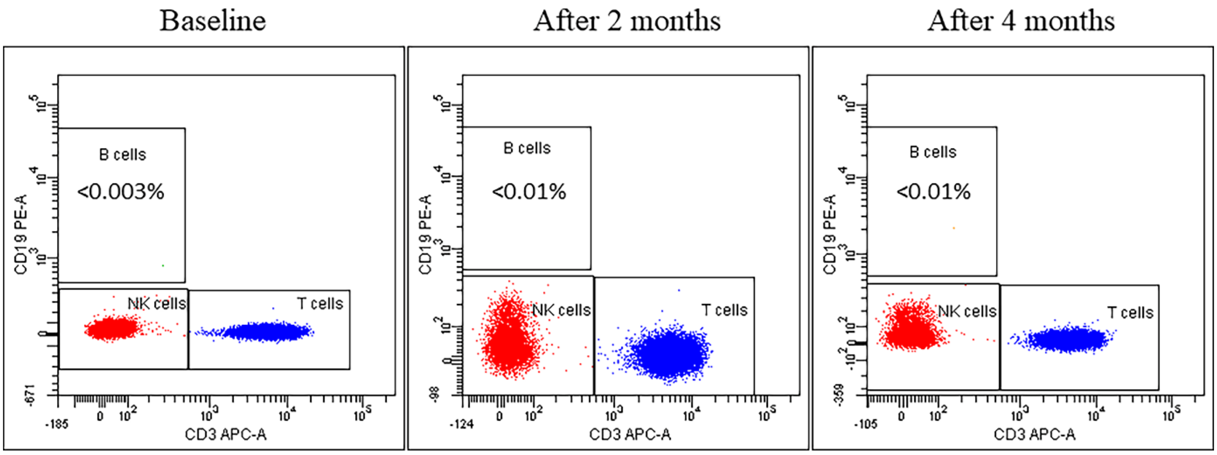

Online Resource 2 Evaluation of the ratio of peripheral B-cells in a COVID-19 patient with hematological malignancy by flow cytometry. Lymphocyte subpopulations were analyzed based on CD19-PE and CD3-APC positivities (Becton Dickinson) and the percentage of B-cells was determined in case of anti-CD20 therapy and after 2 and 4 months on a FASCanto II flow cytometer. No detectable B-cell population was observed at any time point. During the flow cytometric analysis of the samples, 500.000 cells were collected with a sensitivity of 10/500.000, while 100.000 cells were studied in the follow-up samples showing a sensitivity of 10/100.000.
